# Colobome maculaire unilatérale: à propos d’un cas

**DOI:** 10.11604/pamj.2017.28.55.12744

**Published:** 2017-09-21

**Authors:** Meriem El Bahloul, Fouad Chraïbi, Marrakchi Mohammed, Meriem Abdellaoui, Idriss Benatiya

**Affiliations:** 1 Service d’Ophtalmologie, CHU Hassan II, Faculté de Médecine et de Pharmacie de Fès, Maroc

**Keywords:** Colobome maculaire, excavation maculaire, tomographie en cohérence optique, Macular coloboma, macular excavation, optical coherence tomography (OCT)

## Abstract

Le colobome maculaire est une affection congénitale en rapport avec une anomalie de fermeture de la fissure fœtale, pouvant être intégré dans un cadre héréditaire. Il se caractérise cliniquement par une acuité visuelle basse avec une lésion excavée maculaire où le tissu rétinien normal est absent ou rudimentaire et la sclère est ectasique. La tomographie en cohérence optique maculaire est fortement évocatrice du diagnostic et le bilan électrophysiologique s'il est demandé est altéré. Le diagnostic différentiel se pose avec les pathologies entrainant une lésion atrophique et excavée de la macula, particulièrement la toxoplasmose congénitale.

## Abstract (en)

Macular coloboma is a congenital condition characterized by failure of closure of the fetal intraocular fissure which may have a hereditary origin. Clinically, it is characterized by decreased visual acuity with macular excavated lesion characterized by missing or rudimentary retinal tissue and scleral ectasia. Macular OCT strongly supports the diagnosis and electrophysiology examination, if requested, is altered. Differential diagnosis includes pathologies causing atrophic and excavated macular lesion, in particular congenital toxoplasmosis.

## Introduction

La dénomination de colobome s'applique à certaines anomalies congénitales de lœil caractéristiques par leur configuration ou leur siège, et correspondant soit à des fentes ou lacunes où le tissu normal est absent, aplasique ou remplacé par du tissu connectif. Elles sont dues à un défaut de fermeture de la fissure fœtale qui se fait normalement entre la 5^ème^ et la 7^ème^ semaine de vie intra-utérine. Ainsi on peut trouver des fentes qui siègent sur les paupières, l'iris, le cristallin, le corps ciliaire, la choroïde, la rétine et le nerf optique [1]. Les colobomes sont dits typiques lorsqu'ils siègent dans le méridien correspondant au niveau de la fente fœtale soit directement soit dans un méridien un peu plus interne, et ils sont dits atypiques lorsqu'ils sont localisés en d'autres méridiens quelconques, c'est le cas des colobomes maculaires [2]. Nous rapportons l'observation d'un enfant de 10 ans, issu d'un mariage non consanguin, sans antécédent particulier, notamment les antécédents prénataux (une grossesse bien suivie de déroulement normal, menée à terme avec accouchement sans incident).

## Patient et observation

**Figure 1:**
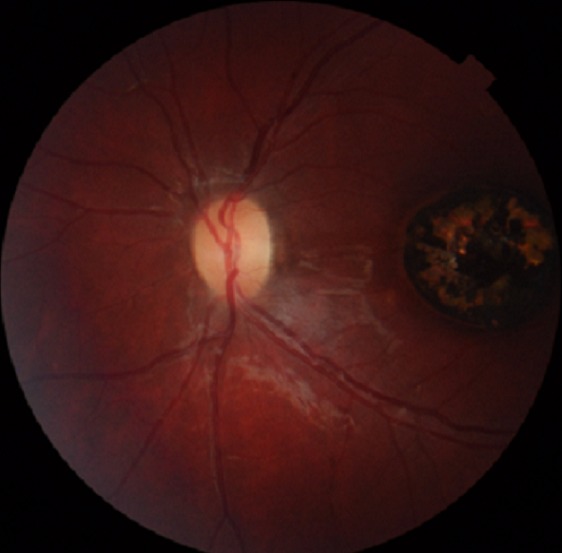
Photo couleur de l’œil gauche objectivant l’aspect du colobome maculaire

**Figure 2:**
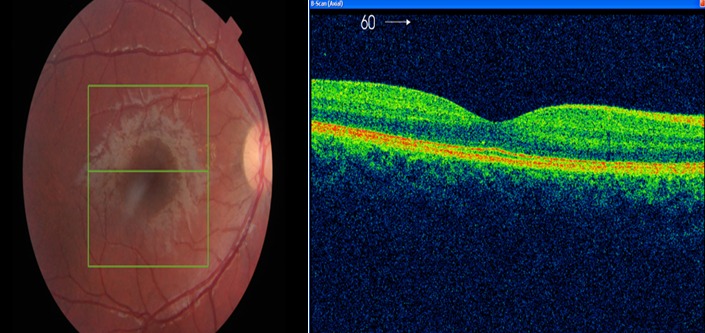
Profil maculaire normal à l’OCT du côté droit

**Figure 3:**
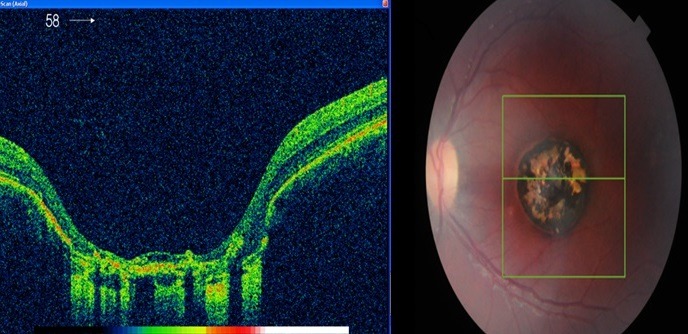
OCT maculaire de l’œil gauche, objectivant une dépression en forme de cratère au niveau de la lésion avec une neurorétine anormalement atrophique et sans nette individualisation de l’épithélium pigmentaire rétinien

L'examen ophtalmologique a mis en évidence une acuité visuelle à 12/10 sans correction au niveau de l’œil droit et à 0.5/10 non améliorable après correction au niveau de l’œil gauche, avec une exotropie de ce dernier. L'examen du segment antérieur était sans particularité au niveau des deux yeux, le fond d’œil était normal à droite, alors qu'il a objectivé au niveau de l’œil gauche une lésion atrophique en plein macula, en forme de cratère, de quatre diamètres papillaires à peu près, bien circonscrite avec présence de pigment en surface et individualisation des gros vaisseaux choroïdiens (Figure 1). L'OCT de la région maculaire était sans particularité à droite (Figure 2), par contre elle a montré à gauche une large dépression accompagnant une rétine neurosensorielle atrophique, sans individualisation nette de l'épithélium pigmentaire rétinien (Figure 3). L'examen général et notamment neurologique était sans particularité, les sérologies de toxoplasmose, rubéole, syphilis et cytomégalovirus étaient négatives. L'examen des parents et de la fratrie n'a mis en évidence aucune anomalie, la sérologie de toxoplasmose réalisée chez la mère était négative. Nous avons retenu le diagnostic de colobome maculaire unilatéral isolé en se basant sur les arguments suivant: l'absence d'antécédents d'épisode infectieux durant la période prénatale, les sérologies négatives ainsi que la négativité de la sérologie de toxoplasmose chez la maman, les caractéristiques cliniques de la lésion et l'absence d'histoire familiale.

## Discussion

Le colobome maculaire est une anomalie de développement d'origine embryologique, ne s'intégrant pas dans un cadre héréditaire quand il est unilatéral et isolé, cependant il existe une forme génétique autosomique dominante d'expression variable et de pénétrance incomplète surtout devant l'atteinte bilatérale [3]. D'autres formes génétiques ont été rapportées et sont souvent liées à des syndromes complexes, notamment le syndrome de sorsby qui est un syndrome malformatif de transmission autosomique dominante, caractérisé par l'association de colobome maculaire bilatéral avec un nystagmus horizontal, une baisse de l'acuité visuelle sévère et des anomalies des mains et des pieds comprenant un raccourcissement des phalanges moyennes et terminales du deuxième au cinquième doigt ou orteil, une hypoplasie ou absence des ongles, les pouces et les hallux sont larges ou bifides, une syndactylie avec des anomalies de flexion des articulations de quelques doigts ou orteils [3]. Cliniquement un colobome maculaire a la forme d'une excavation maculaire de 3 à 6 diamètres papillaires en forme de cratère ou la rétine et la choroïde sont absentes ou rudimentaires, avec une sclérotique ectasique [2, 3]. Sharma et al a prouvé histologiquement sur un cas de colobome maculaire que ces zones manquaient de l'épithélium pigmentaire rétinien et de la choriocapillaire [2, 4]. L'OCT confirment ces données histologiques, l'ERG global s'il est demandé ainsi que le pattern ERG et l'ERG multifocal sont altérés [5]. Le diagnostic différentiel d'un colobome maculaire peut se poser devant une toxoplasmose congénitale, une dystrophie maculaire de la Caroline du Nord, une amaurose congénitale de Leber, une dystrophie des cônes à un stade avancé ou une atrophie aréolaire centrale [6]. La toxoplasmose congénitale est souvent unilatérale, avec des antécédents d'épisode infectieux au cours de la grossesse, une confirmation sérologique est souvent obtenue. Bien que bien limitées, les lésions de toxoplasmose congénitale ne sont pas bien arrondies ou ovales en forme comme un colobome, il existe un risque de réactivation de ces lésions [6]. La dystrophie maculaire de la Caroline du Nord est une maculopathie bilatérale et symétrique à transmission autosomique dominante avec une pénétrance élevée et une expression variable, le stade III de cette maculopathie est caractérisée par d'énorme lésions staphylomateuses maculaires ressemblant à un colobome [7, 8]. L'amaurose congénitale de Leber est une rétinopathie congénitale héréditaire à transmission autosomique récessive, quelques cas sont dominants. Elle se manifeste par un comportement de cécité ou de malvoyance profonde et nystagmus pendulaire, une héméralopie ou une photophobie, une amétropie forte fréquente, avec un aspect au fond d’œil allant de la normale à des lésions atrophiques maculaires pseudocolobomateuses [7]. La dystrophie des cônes et l'atrophie aréolaire centrale sont des rétinopathies héréditaires se traduisant entre la deuxième et la quatrième décade, la lésion maculaire est représenté par une atrophie géographique [6, 7].

## Conclusion

Nous concluons à travers ce cas clinique qu'il ne s'agit pas toujours d'un foyer choriorétinien de toxoplasmose congénitale devant une maculopathie avec large excavation, d'où la nécessité d'une bonne anamnèse et d'un examen clinique détaillé du patient et de sa famille voire le recours à des examens complémentaires pour poser le diagnostic.

## Conflits d’intérêts

Les auteurs ne déclarent aucun conflit d'intérêts.
